# BRD4 modulates vulnerability of triple-negative breast cancer to targeting of integrin-dependent signaling pathways

**DOI:** 10.1007/s13402-020-00537-1

**Published:** 2020-10-02

**Authors:** Yang Zhang, Bingwei Xu, Junfeng Shi, Jieming Li, Xinlan Lu, Li Xu, Helen Yang, Nevean Hamad, Chi Wang, Dana Napier, Shuixiang He, Chunming Liu, Zeyi Liu, Hai Qian, Li Chen, Xiaowei Wei, Xucai Zheng, Jian-An Huang, Olivier Thibault, Rolf Craven, Dongping Wei, Yueyin Pan, Binhua P. Zhou, Yadi Wu, Xiuwei H. Yang

**Affiliations:** 1 Department of Pharmacology and Nutritional Sciences, Department of Molecular and Cellular Biochemistry, and Markey Cancer Center, College of Medicine, University of Kentucky, Lexington, KY USA; 2 Department of Respiratory Medicine, First Affiliated Hospital of Soochow University, Suzhou, Jiangsu Province People’s Republic of China; 3 Department of Oncology, Nanjing Medical University, Nanjing, Jiangsu Province People’s Republic of China; 4 Center of Drug Discovery, China Pharmaceutical University, Nanjing, Jiangsu Province People’s Republic of China; 5 Department of Medical Oncology, the First Affiliated Hospital, Xi’an Jiaotong University, Xi’an, Shanxi Province People’s Republic of China; 6 Department of Statistics, University of Kentucky, Lexington, KY USA; 7 The First Affiliated Hospital of University of Science & Technology of China and Provincial Hospital, Hefei, Anhui Province People’s Republic of China

**Keywords:** Triple-negative breast cancer, Integrin, BRD4, FAK, Targeted therapy, c-Myc

## Abstract

**Purpose:**

Stemming from a myriad of genetic and epigenetic alterations, triple-negative breast cancer (TNBC) is tied to poor clinical outcomes and aspires for individualized therapies. Here we investigated the therapeutic potential of co-inhibiting integrin-dependent signaling pathway and BRD4, a transcriptional and epigenetic mediator, for TNBC.

**Methods:**

Two independent patient cohorts were subjected to bioinformatic and IHC examination for clinical association of candidate cancer drivers. The efficacy and biological bases for co-targeting these drivers were interrogated using cancer cell lines, a protein kinase array, chemical inhibitors, RNAi/CRISPR/Cas9 approaches, and a 4 T1-Balb/c xenograft model.

**Results:**

We found that amplification of the chromosome 8q24 region occurred in nearly 20% of TNBC tumors, and that it coincided with co-upregulation or amplification of c-Myc and FAK, a key effector of integrin-dependent signaling. This co-upregulation at the mRNA or protein level correlated with a poor patient survival (*p* < 0.0109 or *p* < 0.0402, respectively). Furthermore, we found that 14 TNBC cell lines exhibited high vulnerabilities to the combination of JQ1 and VS-6063, potent pharmacological antagonists of the BRD4/c-Myc and integrin/FAK-dependent pathways, respectively. We also observed a cooperative inhibitory effect of JQ1 and VS-6063 on tumor growth and infiltration of Ly6G^+^ myeloid-derived suppressor cells in vivo. Finally, we found that JQ1 and VS-6063 cooperatively induced apoptotic cell death by altering XIAP, Bcl2/Bcl-xl and Bim levels, impairing c-Src/p130Cas-, PI3K/Akt- and RelA-associated signaling, and were linked to EMT-inducing transcription factor Snail- and Slug-dependent regulation.

**Conclusion:**

Based on our results, we conclude that the BRD4/c-Myc- and integrin/FAK-dependent pathways act in concert to promote breast cancer cell survival and poor clinical outcomes. As such, they represent promising targets for a synthetic lethal-type of therapy against TNBC.

**Electronic supplementary material:**

The online version of this article (10.1007/s13402-020-00537-1) contains supplementary material, which is available to authorized users.

## Introduction

Triple-negative breast cancer (TNBC) remains one of the deadliest breast cancer subtypes, as patients carrying this disease are largely dependent on systemic chemotherapies and undergo rapid tumor progression and recurrence [1–3]. TNBC tumors also display a wide spectrum of oncogenic events, including oncogenic mutations (e.g., PI3KCA, K-Ras), epigenetic silencing (e.g., BRCA1) and aberrant copy gain or loss of crucial genes (e.g., c-Myc amplification or PTEN deletion) [4–7]. TNBC is, therefore, a complex disease requiring individualized (targeted) therapies.

There is growing evidence that deregulation of the chromosome 8q24 region denotes a distinct class of TNBC [4, 8]. This genomic anomaly is characterized by a stretch of genes being co-amplified in primary tumors and is frequently accompanied by hyperactivation of the c-Myc oncogene [5, 9]. Being situated at the 8q24.21 locus, c-Myc levels may be elevated in breast tumors through gene amplification or posttranslational modification (e.g., proteasomal regulation) [5, 10, 11]. As a master transcription factor, c-Myc coordinates global gene transcription programs to accelerate tumor growth and disease recurrence [12, 13]. Recently, the pro-tumor activity of c-Myc has been linked to transcriptional and epigenetic regulation by BRD4, a member of the Bromodomain and Extra Terminal (BET) domain-containing family of proteins. Specifically, it was found that c-Myc activity was dependent on interaction between the bromodomain of BRD4 and acetyl groups of transcription factors within RNA polymerase II-organized complexes [13]. This interaction appears targetable by small molecule inhibitors, like JQ1 and BET-762 [14, 15]. Hence, the BRD4-c-Myc axis not only drives tumor development, but also serves as a source of promising targets for mitigating 8q24 amplification-associated malignancy in breast cancer.

Our recent study also argues for a strong role of an integrin-dependent pathway in 8q24 deregulation-linked cancer development [16]. Interestingly, focal adhesion kinase (FAK), an integrin-linked non-receptor tyrosine kinase, is also located within the chromosome 8q24 region (8q24.30) and there is evidence that FAK is amplified and overexpressed in over 40% of breast tumors, and is strongly associated with resistance to chemotherapy [17, 18] and targeted therapies (e.g., anti-PD-L1) [19, 20]. The action of this kinase is tightly associated with several integrin-mediated biological and pathological processes, ranging from cell survival and invasion to DNA damage response and tumor growth and progression [19, 21–23]. These functional roles of FAK are tightly coupled with its Y^397^ autophosphorylation resulting from activation of integrins (e.g., the laminin-binding α3β1 integrin) or their crosstalk with receptor tyrosine kinases (RTKs) [24–26]. This, in turn, activates downstream pathways to affect tumor cell growth or behavior [27, 28]. Hence, targeting the integrin-FAK axis represents another promising avenue for eradicating 8q24 amplification-linked breast cancer.

While the BRD4/c-Myc and the integrin/FAK axes are promising targets for breast cancer [29–31], their functional interactions remain elusive. Also, some of their inhibitors, such as JQ1 (BRD4-c-Myc) or VS-6063 (FAK), have shown limited efficacy in clinical trials, presumably due to the presence of parallel pathways or aberrant activation of downstream effectors [32–35]. These scenarios underscore a need for understanding how these two distinct axes are connected in the clinical setting and at molecular and signaling levels. Here we performed clinical, in vitro and in vivo analyses to assess crosstalk between the BRD/c-Myc axis and the integrin/FAK-dependent pathway in breast cancer and their potential as targets for a synthetic lethal-type therapy. Our data indicate a close clinical and functional alliance of the BRD/c-Myc axis and the integrin/FAK-dependent pathway in TNBC at functional, signaling and clinical levels. Their respective inhibitors, JQ1 and VS-6063, also cooperate in terms of disrupting tumor cell survival, inflammatory microenvironments and growth, thereby representing a reservoir of promising targeted therapies against TNBC.

## Materials and methods

### Cell lines and reagents

Most authenticated breast cancer cell lines were obtained from the ATCC. SUM149, SUM159 and SUM1315 were purchased from Asterand Bioscience (Detroit, MI, USA). Cells were cultured in RPMI-1640 or DMEM (Invitrogen) supplemented with 5–10% FBS (Sigma-Aldrich, St; Louis, MO, USA) at 37 °C in 5% CO_2_. During this study, all cell lines were periodically examined by PCR for Mycoplasma contamination [36]. Sources of most antibodies and chemical inhibitors used have been reported in a prior study [16]. The antibodies directed against BRD4, Ly6G and F4/80 were purchased from Cell Signaling Technology (Danvers, MA, USA) and Abcam (Cambridge, MA, USA), respectively.

### Transfection and expression of ORF constructs, siRNA oligos, shRNAs and CRISPR/Cas9

siRNA oligos for c-Myc, FAK, Bim and β-catenin were obtained from Cell Signaling Technology or Dharmacon (Boulder, Denver, CO, USA), respectively. CRISPR/Cas9-mediated deletion of Snail and Slug was carried out by cloning respective DNA fragments into a gRNA lentiviral plasmid (plasmid #52963, Addgene, Cambridge, MA, USA) and selection for stable Puromycin- and Blasticidin-resistant clones. The sequences of the siRNA oligos and gRNAs used are listed in Table S1. The shRNA for β1 integrin that we used has been described in a prior report [16]. DNA constructs containing the Bim ORF or shRNAs of murine FAK (Plasmid #37015 and #37018) were obtained from Addgene. Cell transfections with siRNA oligos or DNA constructs were conducted using Lipofectamine 2000 (ThermoFisher, Waltham, MA, USA). In addition, tumor cell lines stably expressing β1 integrin or FAK shRNAs were generated via lentiviral infection and subsequent GFP-based sorting by flow cytometry or Puromycin selection.

### Cell viability, cell cycle, apoptosis and signaling assays

Cell viability was evaluated using a MTT assay [16]. For cell cycle analysis, tumor cells were starved overnight, followed by treatment with inhibitors for 36 h in the presence of 10% FBS and, subsequently, stained (10 μg/ml) and analyzed by flow cytometry. To evaluate apoptotic cell death, tumor cells were treated with the indicated inhibitors for 48–72 h in the presence of 5–10% FBS, stained with a combination of propidium iodide and APC-conjugated Annexin V (10 μg/ml, BioLegend), and analyzed by flow cytometry. For analyses of changes in cell signaling, tumor cells were treated with chemical inhibitors or RNA oligos for the indicated time periods, followed by lysis in RIPA buffer in the presence of protease inhibitors and Na_3_VO_4_ [16]. Immunoblotting was carried out by incubating lysates with primary and secondary antibodies, followed by detection using a Chemoluminescence kit (Thermo-Fisher). β-actin was used as loading control. Cell signaling was analyzed in parallel with human phosphor-kinase antibody arrays according to the manufacturer’s instructions (R&D Biosystems, Minneapolis, MN, USA).

### Mouse xenograft and patient TMA analyses

All animal-related studies were conducted under the guidelines and regulations of the University of Kentucky and were approved by the institutional IACUC review board. For this study, Balb/c mice were purchased from Charles River Laboratories (Boston, MA, USA). 4 T1 cells were injected at 1 × 10^7^ per mouse into mammary fat pads, followed by treatment with vehicle (peanut oil 100 μl) alone or inhibitors in oral gavage. Tumor sizes were measured using a caliper twice per week. At the end of the study, the mice were euthanized and tumors and internal organ samples were collected. Tissue microarrays (TMAs) harboring paraffin-embedded breast tumor samples were constructed using a patient cohort recently diagnosed or treated at the University of Kentucky-Chandler Medical Center and obtained through the Biospecimen core. Antibody staining was scored and statistically analyzed as described before [16].

### Data mining of TCGA patient cohort and statistical analyses

Gene amplification and mRNA expression levels in the breast cancer patient cohort present in the TCGA database were assessed using the c-BioPortal platform [7]. A two-sample t-test was used to assess differences in tumor size or weight between groups. ANOVA and Holm’s procedure were applied for multiple group comparisons and adjustments. Kaplan-Meier curves and Logrank tests were employed to compare survival times among groups, and the Turkey-Kramer method was used for making multiple comparison adjustments.

## Results

### Genomic deregulation and altered expression of FAK and c-Myc in TNBC patients

**Fig. 1:**
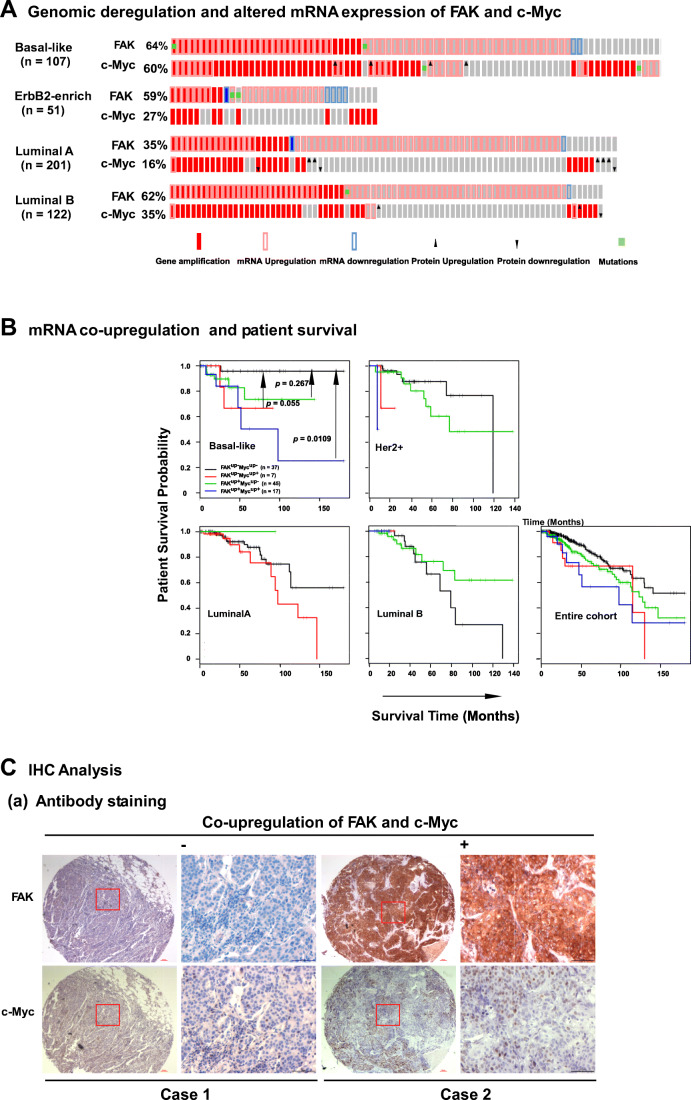

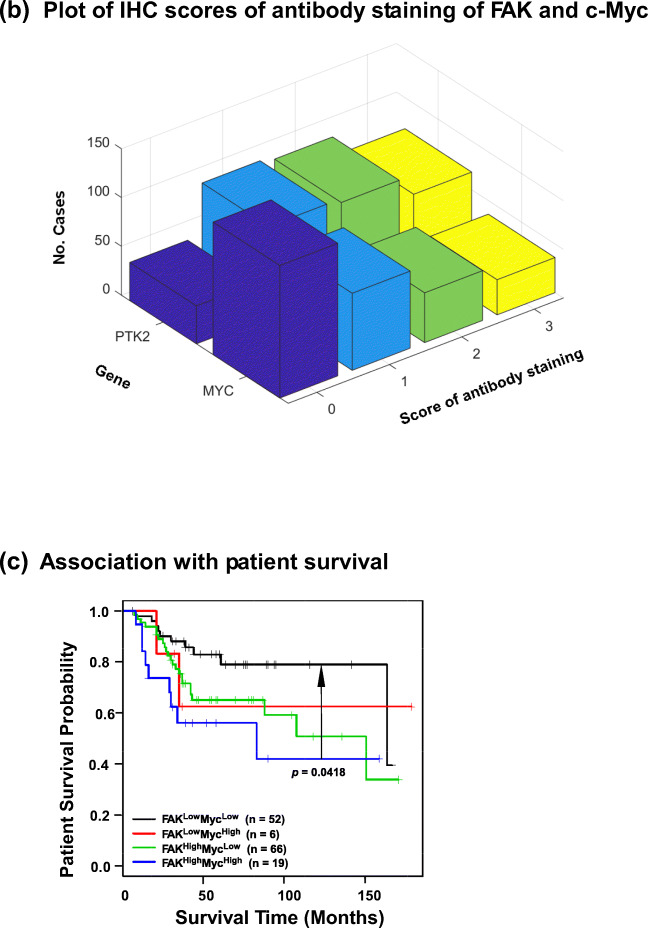
Genomic and expression alterations of FAK and c-Myc in primary tumors of the TCGA database and a local breast cancer patient cohort. **A**-**B** Genomic and expression analyses of FAK and c-Myc in the PAM50 basal-like subtype in the TCGA patient cohort. mRNA alterations were assessed using RNA sequence-based data and a Z-score cut-off value of 2.0-fold. **A** Primary tumors exhibiting alterations in gene copy number/amplification and mRNA upregulation or downregulation are indicated in solid and hollow red or blue bars, respectively. The cut-off value for mRNA upregulation was set at 2.0. Green square: gene mutation. Dark arrows: Changes in protein expression. **B** Plot of patient survival probability over time. *Y-axis*, probability of patient survival; *X-axis*, duration of patient survival (months). Patients from four subtypes or the entire cohort were stratified according to upregulation of FAK and c-Myc mRNA, alone or both (FAK^UP-^MYC^UP-^, FAK^UP+^MYC^UP-^, FAK^UP-^MYC^UP+^ and FAK^UP+^MYC^UP+^), and analyzed statistically. The number of patients and Turkey-Kramer *p* values are indicated for the basal-like subtype only and not for the rest of the subtypes due to lack of effective stratification or meaningful comparisons between subgroups. **C** Association between co-overexpression of FAK and c-Myc and patient survival in a local TNBC patient cohort (*n* = 165). (a) Representative images of antibody staining of primary tumors with concurrent null/low (Case 1) or high (Case 2) expression of FAK and c-Myc are shown in low (left) and high (right) magnification. Scale bar: 100 μm. (b) Plot of antibody staining intensity of FAK and c-Myc in the patient cohort. (c) Plot of patient survival probability over time. The entire TNBC population in the patient cohort was stratified into four groups by FAK and c-Myc protein expression. Turkey-Kramer *p* values were calculated for all subgroups. The *p* value for the difference between the FAK^High^MYC^High^ and FAK^Low^MYC^Low^ groups is indicated

We initially interrogated clinical associations of FAK and c-Myc, which are frequently deregulated at the genomic level in breast tumors [19, 37]. Our analysis of the TCGA breast cancer patient cohort (*n* = 816) revealed that FAK or c-Myc were altered at the genomic level in 16–60% of primary tumors across four subtypes (Fig. 1A, Table S2) [38]. The c-Myc and FAK loci are adjacently situated in the chromosome 8q24 region (8q24.21 and 8q24.30, respectively), and their elevated mRNA expression correlated with a poor patient survival (Fig. S1A), consistent with recent studies [17, 39]. In addition, we found that FAK exhibited an association between genomic amplification/copy number gain and mRNA expression (Fig. S1B). These data suggest a strong link between 8q24 region-based malignancy and co-upregulation of FAK and c-Myc. We also noted a high concordance between FAK and c-Myc gene copy number gain/amplification (Table S2). The Spearman correlation values appeared in an order of Luminal B > Luminal A > ErbB2-enriched subtypes. Strikingly, we found that only patients with basal-like/TNBC subtypes exhibiting co-upregulation of these two genes (FAK^UP+^MYC^UP+^) displayed poorer survival rates than their counterparts (FAK^UP-^MYC^UP-^) (50 vs ~150 months, *p* = 0.0109) (Fig. 1B). In contrast to FAK, NDRG1 and AGO2, which also reside in the 8q24 region [40], exhibited minimal associations with c-Myc (Fig. S1,c). Furthermore, our IHC analysis revealed a strong co-upregulation of FAK and c-Myc at the protein level (Fig. 1C, a, b), as reflected by the Spearman correlation value (0.328, *p* < 0.0001). In this independent TNBC cohort, patients with co-upregulation (case #2, FAK^High^MYC^High^) also exhibited a significantly shorter survival duration than their counterparts (case #1, FAK^Low^MYC^low^) (*p* = 0.0402) (Fig. 1C, c). Collectively, these clinical data indicate a strong association between FAK/c-Myc co-expression and malignancy of TNBC.

### Functional link between FAK and c-Myc in TNBC cells

**Fig. 2:**
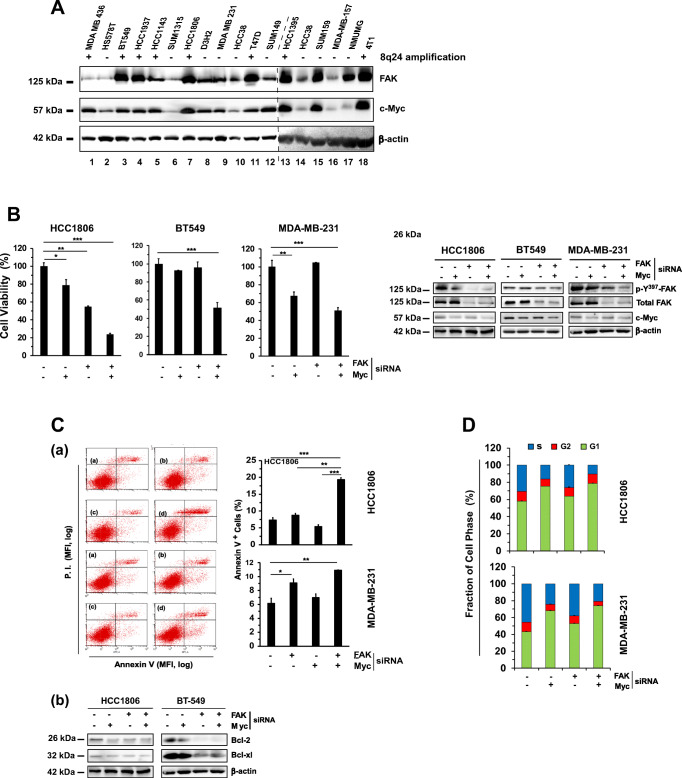
Co-amplification, co-overexpression and functional interaction of FAK and c-Myc across TNBC cell lines. **A** Expression profile of FAK and c-Myc proteins across a panel of human basal-like/TNBC cell lines. Tumor cells were lysed in RIPA buffer and immunoblotted. 4 T1, a mouse basal-like tumor cell line and two luminal cell lines (murine NMuMG and human T47D) were included for comparison. **B**-**D** Effect of simultaneous FAK and/or c-Myc knockdown. TNBC cell lines with (HCC1806 and BT549) or without (MDA-MB-231) co-amplification of FAK and c-Myc were treated with siRNA oligos for 24 h and subsequently analyzed for cell viability by MTT assay. The efficiency of protein knockdown was assessed by Western blotting (**B**). Analysis of apoptotic cell death (**C**): (a) plots of mean fluorescence intensity (MFI) of propidium iodide (PI) and Annexin V antibody staining. Right panel, percentages of gated Annexin V^+^ cells (mean ± SEM, *n* = 3). (b) Expression analysis of major pro-apoptotic proteins by Western blotting. **D** Changes in proportion of cell cycle phases in HCC1806 and MDA-MB-231 cells. *p* values: *: *p* < 0.05; **: *p* < 0.005; ***: *p* < 0.001

We next investigated the functional significance of FAK and c-Myc co-upregulation in the TNBC subtype. We found that FAK and c-Myc were co-overexpressed at the protein level in nearly half of the 16 TNBC cell lines examined (Fig. 2A), thereby recapitulating their deregulation in the clinical setting (Fig. 1). This co-overexpression coincided with amplification/copy number gain of the chromosome 8q24 region in some of the TNBC cell lines, including HCC1806, BT549 and SUM159 (Table S3), based on analysis of the relevant dataset at the cBioportal site [38]. In addition, the level of total FAK protein in this group was 3-fold higher than in their counterparts (HCC38 and MDA-MB-157) (Fig. 2A, Table S3). Interestingly, we detected a similar co-upregulation in the murine 4 T1 line, a widely adopted model for dissecting TNBC malignancy (Fig. 2A). A similar trend was detected in MDA-MB-231 cells, which are known to exhibit oncogenic activation of K-Ras and B-Raf. Furthermore, we found that simultaneous downregulation of FAK and c-Myc via RNAi synergistically decreased the viability of two of the cell lines harboring 8q24 amplifications, HCC1806 and BT-549, compared to the control cell line MDA-MB-231 (Fig. 2B). This effect was also mirrored by a differential impact on apoptotic cell death, as indicated by a > 2-fold increase in the proportion of Annexin V^+^ cells, and a decrease in the levels of anti-apoptotic Bcl2 and Bcl-xl in HCC1806, but not MDA-MB-231 cells (Fig. 2C). In addition, the simultaneous downregulation led to a > 2-fold decrease in cell cycle progression towards the S phase, regardless of the copy number status of the 8q24 region (Fig. 2D). Combined, these data indicate that FAK and c-Myc cooperatively promote tumor cell proliferation and survival related to 8q24 amplification in the TNBC subtype.

### Susceptibility of TNBC cells to pharmacological inhibition of FAK and the BRD4/c-Myc axis

**Fig. 3:**
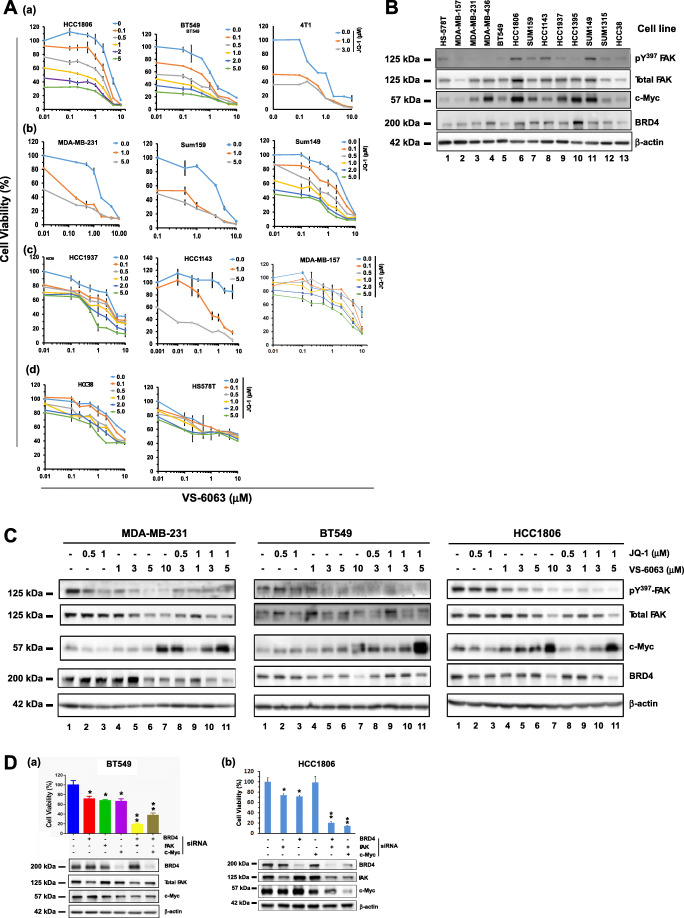
Functional effects of the combination of chemical inhibitors of FAK and the BRD4/c-Myc axis in TNBC cells. **A** Profile of TNBC cell sensitivity to VS-6063 and/or JQ1. A panel of representative TNBC cell lines varying in 8q24 deregulation-associated gene amplification (Supplementary Table S3, Fig. 2) was treated with varying doses and combinations of VS-6063 and JQ1 for 72 h, and subsequently, analyzed for cell viability. Values were calculated as % of DMSO control (mean ± SEM, *n* = 3). **B** Levels of active and total FAK, c-Myc and BRD4 across a panel of representative TNBC cell lines. Tumor cells were lysed in siRIPA buffer and immunoblotted for the indicated proteins. **C** Analyses of dose- and time-dependent effects of VS-6063 and JQ1 in multiple TNBC cell lines (HCC1806, MDA-MB-231 and BT549). Tumor cells were treated with DMSO (control) or varying doses of inhibitors for 24 h, lysed in RIPA buffer, and analyzed for the indicated proteins by Western blotting. **D** Effect of siRNA oligo-mediated downregulation of FAK, c-Myc and BRD4 in HCC1806 (a) and BT549 (b) cells. Tumor cells were subjected to RNA-mediated knockdown, followed by examination of cell viability using a MTT assay (top panel), and knockdown efficiency by Western immunoblotting (bottom panel). Values were calculated as % of DMSO control (mean ± SEM, *n* = 3). *p* values: *: *p* < 0.05; **: *p* < 0.005; ***: *p* < 0.001

With the prevalence of deregulation of the chromosome 8q24 region across the TNBC subtype (Fig. 2), we sought for a corresponding pharmacological targeting strategy. Since FAK and c-Myc are susceptible to inhibition by small molecule inhibitor VS-6063, or indirectly by JQ1, an inhibitor of BRD4 [41], we assessed the response of TNBC cells to these two inhibitors (Fig. 3A). We found that the panel of representative TNBC cell lines tested could be divided into four groups in terms of cell viability: (*a*) high sensitivity to both inhibitors, (*b*) high and intermediate sensitivity to VS-6063 and JQ1, respectively, (*c*) being sensitive to the inhibitor combination only and (*d*) being resistant to the individual inhibitors or their combination. Nearly 75% of the TNBC cell lines tested were responsive to co-targeting of FAK and c-Myc by chemical inhibitors. The additive or synergistic effects appeared to fall within the range of 0 < JQ1 < 0.5 μM and 0.5 μM < VS-6063 < 10.0 μM (Fig. 3A). Consistent with these observations, the inhibitor sensitivity of the TNBC lines seemed to correlate with the expression or activation status of FAK and c-Myc or BRD4 (Figs. 2A, 3A-B). In addition, we found that the sensitivity of TNBC cells to the VS-6063/JQ1 combination was supported by the effect of co-downregulation of FAK and c-Myc or BRD4 by RNAi (Fig. 3D). Notably, downregulating BRD4 by RNAi was equally effective to that of c-Myc with respect to suppression of cell viability in both HCC1806 and BT549 cells. The collaboration of VS-6063 and JQ1 in inhibiting TNBC cell viability was supported by a JQ1-based screening, where VS-6063 was superior to inhibitors of other kinases or pathways with respect to the JQ1 collaboration (Fig. S2). Collectively, these results suggest a genomic alteration-linked functional cooperation between FAK and c-Myc in TNBC cells. Intriguingly, we observed dose- and time-dependent changes in FAK activity and BRD4, but not c-Myc, in multiple TNBC cell lines, as indicated by a decreased Y^397^ phosphorylation (Fig. 3C). Moreover, a cooperative anti-tumor effect of JQ1 and VS-6063 was detected with additional FAK inhibitors, including VS-6062 or TAE226 (Fig. S3). The on-target effect of VS-6063 was supported by our analysis of β1 integrin-deficient MDA-MB-231 and HCC1143 cells (Fig. S4).

### Link between co-inhibition of FAK and the BRD4/c-Myc axis and Bim-associated cell death

**Fig. 4:**
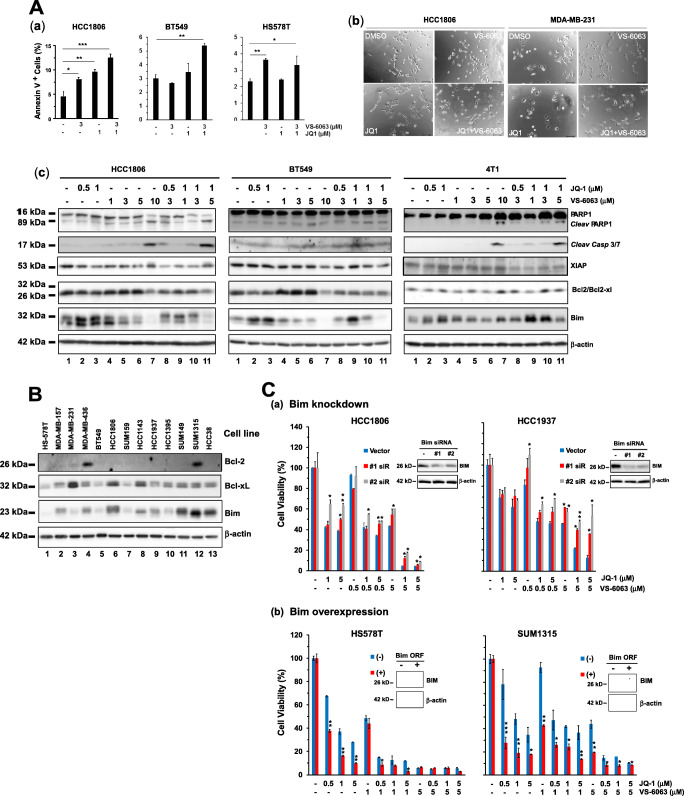
Link between co-inhibition of FAK and the BRD4/c-Myc axis and cell survival. **A** Pro-apoptotic effect of VS-6063 and JQ1. (a) Induction of apoptotic cell death. Percentages of gated Annexin V^+^ cells (mean ± SEM, *n* = 3). (b) Changes in cell morphologies. HCC1806 and MDA-MB-231 cells were treated with 3 μM VS-6063 or 1.0 μM JQ1 alone or in combination for 24 h and imaged microscopically. Scale bar: 50 μm. (c) Changes in major pro- and anti-apoptotic proteins. Tumor cells were treated with inhibitors for 36 h, lysed in RIPA buffer, and immunoblotted for cleaved PARP1, Caspase 3/7 (4 T1/HCC1806 lines), Bcl2, Bcl-xl, XIAP and Bim. **B** Expression profile of endogenous Bim, Bcl2 and Bcl-xl proteins across a panel of representative TNBC cell lines. **C** Direct role of Bim in tumor cell sensitivity to VS-6063 and JQ1. Tumor cells were treated with siRNA oligos or transfected with Bim ORF, followed by analysis of differences in cell viability (Values: mean ± SEM, *n* = 3). (a-b) Effect of Bim knockdown or overexpression on cell viability. Tumor cells were transiently transfected with control and Bim RNAi oligos or cDNA/ORF constructs, followed by 48 h of culture prior to the MTT assay or lysing in RIPA buffer

We next determined if there was a link between the cooperative anti-tumor effect of VS-6063 and JQ1 and apoptosis. As shown in Fig. 4A, the inhibitors exhibited a strong cooperative effect in terms of induction of apoptotic cell death in the HCC1806 and BT549 lines only, consistent with our prior RNAi-based analysis (Fig. 2C). This observation was also corroborated by our microscopic imaging-based analysis (Fig. 4A, b). Notably, treatment with VS-6063 alone or in combination with JQ1 led to a marked increase in the number of floating or dying cells in the HCC1806 line, but not in the MDA-MB-231 line. In addition, the tumor cells exhibited flattened morphologies in both lines, reminiscent of a phenotype associated with cell cycle arrest or senescence. Together, these data suggest that co-inhibition of FAK and c-Myc cooperatively impairs cell survival in a subgroup of TNBC.

Furthermore, we observed corresponding dose-dependent changes in the cleavage of PARP1 and the expression of Caspase 3/7, as well as in levels of apoptosis mediators, including Bcl-2, Bcl-xl, XIAP and Bim (Fig. 4A, c). The cell lines exhibiting a higher sensitivity to the combination of VS-6063 and JQ1 (i.e., HCC1806 and SUM149) appeared to express higher levels of Bim or to exhibit higher ratios of Bim/(Bcl2 + Bcl-xl) than their counterparts (e.g., HS-578 T) (Fig. 4A, b). Attenuating Bim levels via siRNA decreased the sensitivity to VS-6063 and/or JQ1 in both the HCC1806 and HCC1937 cell lines (Fig. 4C, a). Conversely, we found that overexpression of Bim in pY^397^-FAK-low cell lines, including Hs578T or SUM1315, markedly increased the sensitivity to these inhibitors (Fig. 4C, b). These data indicate that FAK and c-Myc synergistically drive tumor cell survival largely by decreasing the cellular level of Bim or the ratios of Bim/Bcl2 + Bcl-xl, regardless the endogenous level of Bim.

### Key signaling effectors upon co-targeting of FAK and the BRD4/c-Myc axis

**Fig. 5:**
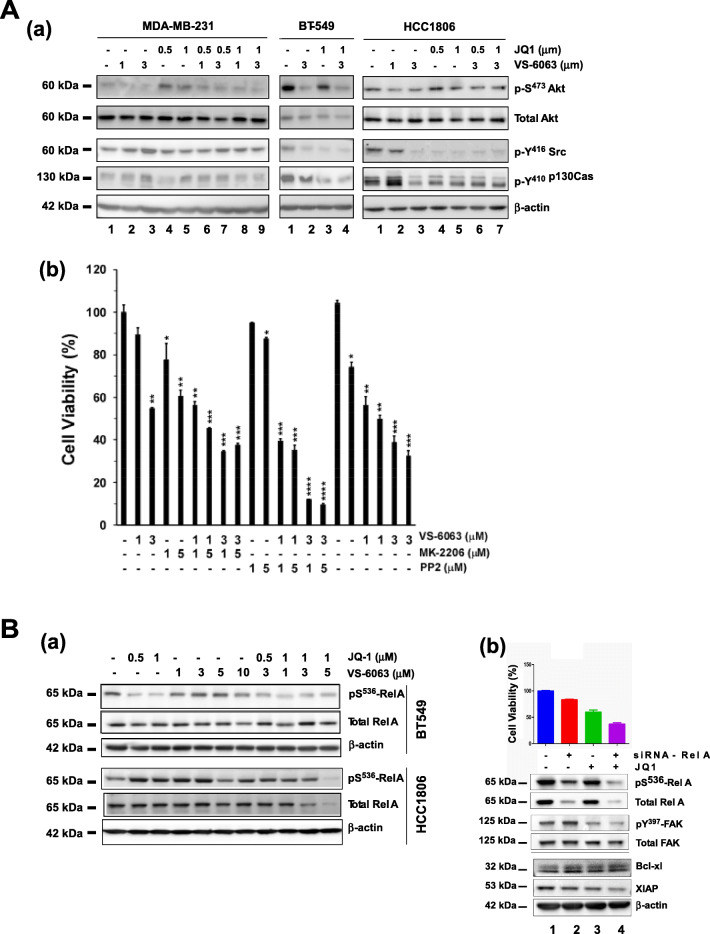
Signaling basis of the cooperative effect of co-inhibition of FAK and c-Myc in TNBC cells. **A** (a) Combined effect of VS-6063 and JQ1 on cell signaling in MDA-MB-231, BT-549 and HCC1806 cells. The respective cells were treated with inhibitors for 24 h, followed by lysis in RIPA buffer and immunoblotting. (b) Combined effect of FAK inhibitor VS-6063 and inhibitors of Akt or c-Src in HCC1806 cells. Cells were treated with inhibitors for 72 h prior to viability assays. **B** Effect of inhibitor treatment or siRNA-mediated knockdown on the NF-κB pathway. TNBC cells (BT549 and HCC1806) were treated with the indicated doses of inhibitors or a combination of inhibitor and siRNA oligos of Rel A (a), followed by Western blot analysis of total and phosphorylated Rel A (a) or cell viability assessment using a MTT assay (b). Values: mean ± SEM (*n* = 3). *p* values: *: *p* < 0.05; **: *p* < 0.005; ***: *p* < 0.001

We subsequently attempted to dissect the common signaling pathways regulated by FAK and c-Myc in TNBC cells. Our analysis showed that inhibiting FAK or c-Myc alone or in combination with VS-6063 and JQ1 gave rise to an inhibitory effect on Src/p130Cas complex-dependent signaling in BT-549 and HCC1806, two cell lines exhibiting 8q24 deregulation, as reflected by >80% reduction in the phosphorylation of Y^416^ and Y^410^ residues in c-Src and p130Cas, respectively, compared to that in MDA-MB-231 cells (Fig. 5A). Furthermore, we found that the BT549 cell line, which is known to be PTEN deficient [42], exhibited a marked inhibition in cell survival-linked S^473^ phosphorylation of Akt compared to that in either HCC1806 or MDA-MB-231 cells (data not shown). Consistent with this observation, in the HCC1806 cell line VS-6063 exhibited a strong collaboration with PP2, an inhibitor of Src family kinases, but only a modest cooperation with MK-2206, an Akt inhibitor (Fig. 5A, b). In addition, we noted a concomitant decrease in Pinch (data not shown), which is implicated in Src-mediated cell survival [30, 43].

Given the susceptibility of the NF-KB-associated pathways to BRD4-dependent epigenetic regulation [1, 44], we next set out to examine their involvement in tumor cell responses to co-inhibition of integrin/FAK-dependent signaling and BRD4. We found that in the BT549 cell line, inhibiting the activity or expression of FAK and c-Myc via RNAi or specific inhibitors impaired S^536^ phosphorylation of RelA/p65 (Fig. 5B, a, b). A similar effect was observed on cell viability or level of XIAP upon RNAi oligo-mediated silencing of RelA/p65 or JQ1 treatment. In contrast, in the HCC1806 and SUM159 cell lines, inhibitor treatment only decreased the level of total RelA/p65, not its phosphorylated form (Fig. 5B). Combined, these data indicate that chemical inhibitor-mediated co-targeting of FAK and the BRD4/c-Myc axis in TNBC cells is largely associated with inhibition of the PI3K/Akt- and c-Src/p130CAS-dependent pathways, and to a lesser extent the RelA/p65-driven NF-ĸB network.

### Link between BRD4 and addiction to the integrin/FAK axis

**Fig. 6:**
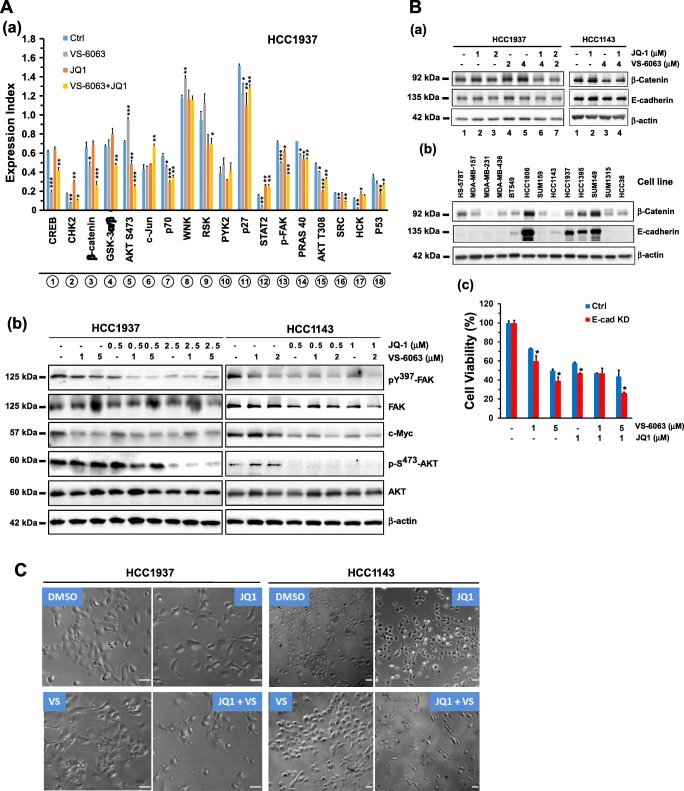

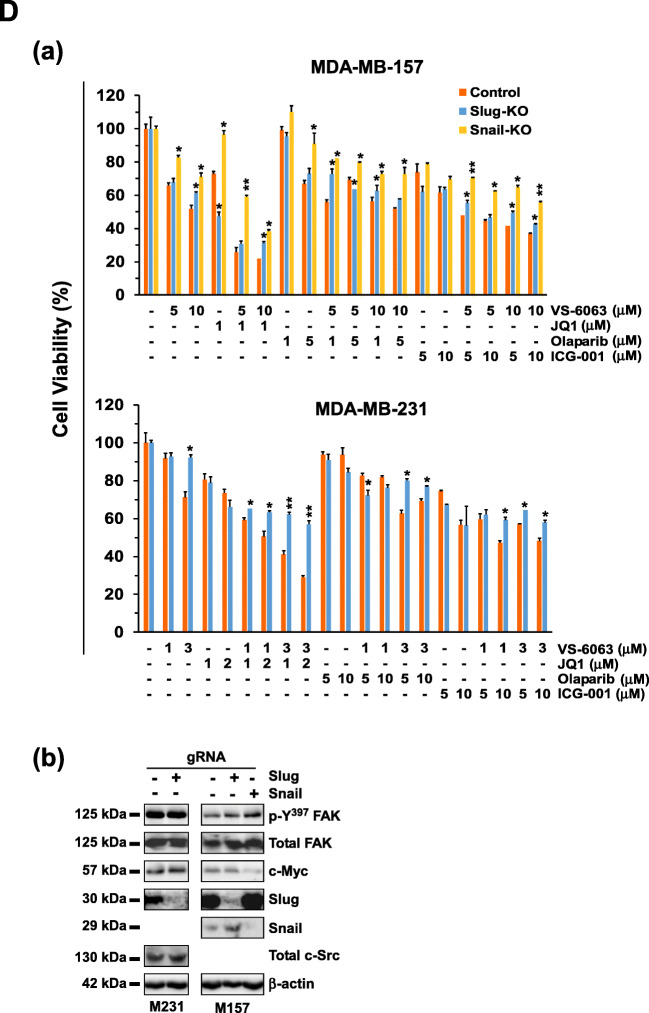
BRD4-associated regulation of tumor cell vulnerability to targeting integrin/FAK-dependent signaling. **A** Screening for signaling pathways or regulators affected by VS-6063 and JQ1 using a protein kinase antibody array. (a) HCC1937 cells were treated with 3.0 μM VS-6063 and/or 1.0 μM JQ1 for 24 h prior to Western blot analysis. The relative expression levels or activation intensities of protein kinases or transcription factors were determined by measuring the optical intensities of bands (in duplicate) on the array using Image J software. Values: mean ± SEM (*n* = 2). (b) Validation of changes in activities or expression levels of individual signaling molecules by Western blotting of cell lysates. **B** Link between cell-cell adhesion and tumor cell sensitivity to VS-6063 and JQ1. (a-b) Expression of E-cadherin and β-catenin upon inhibitor treatment across a panel of TNBC cell lines. (c) Effect of E-cadherin knockdown on the viability of HCC1937 cells (mean ± SEM, *n* = 3). Expression index (mean ± SEM, *n* = 2) calculated from the OD values of dentistry-based analyses. **C** Induction of an EMT-like phenotype in HCC1937 and HCC1143 cells by VS-6063 and JQ1. Tumor cells were treated with control (DMSO) or 3.0 μM VS-6063 and/or 1.0 μM JQ1 for 6 or 24 h, and imaged microscopically. *p* values: *: *p* < 0.05; **: *p* < 0.005; ***: *p* < 0.001. **D** Link between the EMT-inducing transcription factors Snail/Slug and tumor cell susceptibility to FAK and c-Myc inhibitors. Top panel (a) effect of Snail and Snail knockout cell viability (mean ± SEM, *n* = 3). Tumor cells varying in expression of gRNAs of Snail or Slug were treated with inhibitors for 72 h, and analyzed for viability (mean ± SEM, *n* = 3). Bottom panel (b), validation of CRSIPR/Cas9-mediated deletion of Slug and Snail protein by Western blotting. *p* values: *: *p* < 0.05; ** *p* < 0.005; ***: *p* < 0.001

The availability of a subset of TNBC cell lines (HCC1937 and HCC1143) that were sensitive to the combination of VS-6063 and JQ1, but not to individual inhibitors (Fig. 3A, c), provided a unique window for delineating resistance to co-targeting of the integrin-FAK and the BRDX4/c-Myc axes. For this purpose, we conducted a screening analysis using a protein kinase antibody array. We found that the combination of VS6063 and JQ1 cooperatively impaired activation of FAK and its downstream signaling through the Akt/mTOR or the Src/p130-dependent pathways (Fig. 6A, Fig. S5). These effects were validated by Western blotting for individual signaling mediators (Fig. 6).

We also detected a synergistic or additive decrease in β-catenin level upon combined treatment with inhibitors (Fig. 6A). In line with this observation, silencing of E-cadherin increased sensitivity to the combination of VS-6063 and JQ1 (Fig. 6B, b-c). Furthermore, we found that JQ1 treatment led to an epithelial-mesenchymal transition (EMT)-like morphological change (Fig. 6C). Given the widely recognized role of Slug and Snail in EMT induction in breast cancer cells [45], we suspected that the JQ1-mediated phenotype might be associated with activation of these transcription factors. We tested this option by examining the effect of Snail or Slug deletion via the CRISPR/Cas9 approach on tumor cell response to the combined VS-6063 and JQ1 treatment. We found that knockout of Slug or Snail dampened tumor cell sensitivity to JQ1 or its combination with VS-6063 in both MDA-MB-157 and MDA_MB-231 cells (Fig. 6D). By comparison, such knockouts had minimal effects on tumor cell responses to inhibitors of PARP1 (Olaparib) or CBP of the Wnt pathway (ICG001). Together, these data suggest that tumor cell sensitivity to co-inhibition of FAK and the BRD4/c-Myc axis is linked to the EMT program and its associated transcription factors.

### In vivo effects of pharmacological inhibitors of the BRD4/c-Myc and integrin/FAK axes

**Fig. 7:**
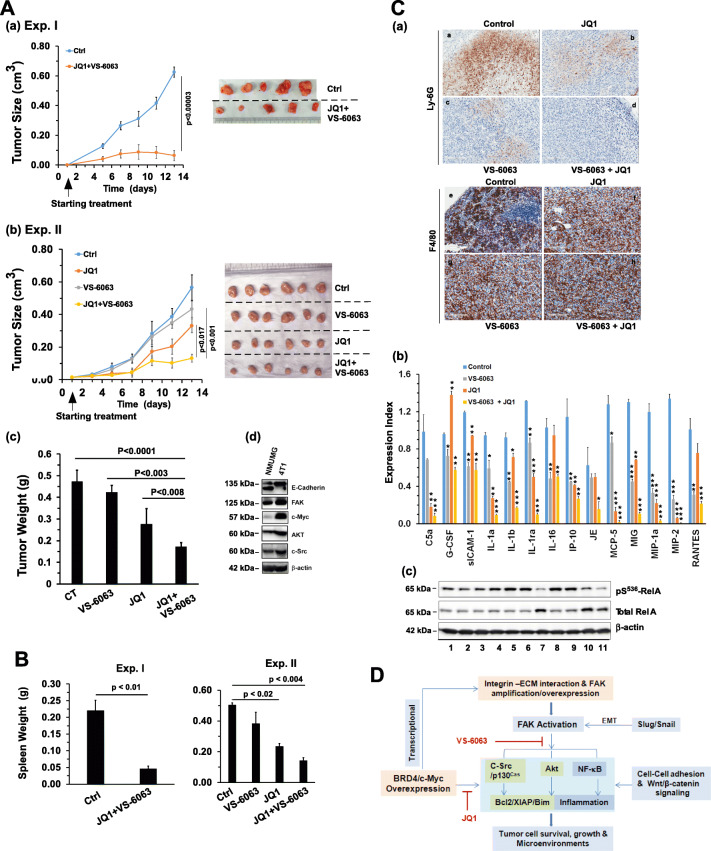
In vivo effect of VS-6063 and JQ1-mediatetd co-inhibition of FAK and the BRD4/c-Myc axis. **A** Effect on tumor growth. 6–7-week-old Balb/c mice were individually injected with 4 T1 cells and treated with vehicle control (peanut oil) or the indicated doses of inhibitors. Tumor volumes (cm^3^) were calculated by length x width x height × 0.52. Mice were treated with a combination 75 mg/kg VS-6063 and 25 mg/kg JQ1 in Experiment I (a), and 50 mg/kg VS-6063 or 25 mg/kg JQ1 alone or in combination in Experiment II (b). Number of mice per group: *n* = 4–8. (c) Verification of expression of key signaling molecules in 4 T1 cells. NMuMG and 4 T1 cells were lysed and immunoblotted with antibodies directed against the indicated proteins. **B** Effect on splenomegaly. The average weight per group is shown (mean ± SEM, *n* = 4–8). **C** Link to tumor microenvironments. (a) IHC images of Ly6G and F4/80 antibody-stained tumor tissues from Experiment II. (b) Expression of cytokines in tumor tissues. Tumor tissues were lysed in RIPA and homogenized, after which supernatants were analyzed using murine cytokine antibody arrays. (c) Effect of VS-6063 and JQ1 on the NF-kB pathway. 4 T1 cells were treated with inhibitor for 24 h, lysed in RIPA and analyzed for the indicated signaling molecules by Western blotting. The *p* values obtained from analyses of differences between treatments are indicated. **D** A working model for functional and signaling cooperation of FAK and c-Myc in breast cancer

Given the strong in vitro effect of co-targeting of the integrin-FAK and BRD4c-Myc axes, we next evaluated the anti-tumor efficacy of co-inhibition of these pathways in vivo*.* Given the effect of the inhibition in 4 T1 cells (Fig. 2), a mouse-based syngeneic model was adopted. We found that the combination of VS-6063 and JQ1 markedly decreased the tumor volumes in mice over a two-week period (*p <* 0.001), compared to the control group in both sets of experiments (*p* = 0.001 and 0.016 for Exp. I and II, respectively) (Fig. 7A). A similar decrease was noted for tumor weights (*p* = 0.0001 in Exp. II). VS-6063 and JQ1 also cooperatively inhibited or diminished 4 T1 cell-induced splenomegaly in tumor-bearing mice (Fig. 6B).

After IHC analysis, we also detected a marked decrease in Ly6G^+^ infiltrating myeloid-derived suppressor cells (MDSC) in tumor stroma, but not in F4/80^+^ macrophages (Fig. 7C, a). In addition, our cytokine antibody array-based analysis showed that VS-6063 and JQ1 cooperatively reduced MDSC-associated cytokine levels in tumors, including C5a, IL1α, IL1β, MCP-5, MIG, MIP-1a, MIP-2 and RANTES (Fig. 7C, b). Also, a cooperative effect of VS-6063 and JQ1 on the NF-κB pathway was observed, as indicated by a decreased S^536^ phosphorylation of RelA/p65 (Fig. 7C, c). Together, these in vivo observations indicate that co-inhibition of FAK and the BRD4/c-Myc axis effectively suppresses tumor growth and immunity evasion-oriented microenvironments.

## Discussion

Here we attempted to dissect crosstalk between the integrin/FAK-dependent signaling pathway and the BRD4/c-Myc axis in the TNBC subtype. Our analyses showed that these two axes are frequently co-deregulated in primary breast tumors and are significantly associated with poor patient survival. The importance of this clinical association is underscored by a strong cooperation between FAK and the BRD4/c-Myc axis in driving tumor cell proliferation, survival and signaling via multiple pathways. The pharmacological co-inhibition of these two axes also impairs tumor growth and its microenvironments in vivo. As a result, our study opens up a new possibilities for the treatment of malignant (TNBC) breast cancer (Fig. 7D).

### Genetic basis of FAK/c-Myc co-amplification/co-overexpression

FAK, c-Myc, and the associated molecular and signaling networks have long been regarded as key drivers of breast tumorigenesis [19, 46]. Our results indicate that these molecular drivers or networks are frequently co-deregulated and contribute to a poor survival in the TNBC subgroup. In addition, an association between the co-amplification of FAK and c-Myc and patient survival is expected, since each of these genes is known to be associated with breast tumorigenesis [19, 47, 48]. A similar phenomenon has been described for two other 8q24 amplification-linked genes, AGO2 and NDRG1 [16, 19, 23]. Mechanistically, this phenomenon is likely related to the complex regulation of c-Myc expression. The mRNA expression of c-Myc has been found to be influenced by the copy numbers of its neighboring genes within the 8q24 region, particularly the *PVT1* gene [49]. In addition, c-Myc frequently undergoes posttranslational regulation, including ubiquitination-based proteasome degradation [49, 50]. It is worth noting that 8q24 deregulation appears to occur at a high rate in a subset of breast tumor cell lines with a metaplastic- squamous cell-like morphology and an invasive and inflammatory phenotype (HCC1806, BT549 and 4 T1) [51, 52]. This notion is consistent with a high level of total FAK in these cell lines (Fig. 2A), which appears to correlate with an invasive or motile phenotype of tumor cells [53]. In addition, BT549 cells may possess a high level of active or pY^397^FAK due to PTEN loss, compared to MDA-MB-231 cells [42]. This scenario is consistent with the altered signaling link between FAK and NF-κB in the absence of active PTEN [54]. If the coupling of genetic alterations and aggressiveness of TNBC can be proven in a large patient cohort, the VS-6063/JQ-mediated co-disruption of the integrin-FAK and BRD4-c-Myc axes should have an immediate impact on the clinical treatment of this disease.

### Cooperation of the integrin/FAK and the BRD4/c-Myc axes at the cellular level

The clinical importance of the co-deregulation of FAK and the BRD4/c-Myc axis is corroborated by their cooperative role in cell survival. This function appears to be strongly linked to the level of mitochondria-linked anti-apoptotic Bim, consistent with recent studies on BRD4 [55, 56]. At the signaling level, the alliance of FAK and the BRD4/c-Myc axis appears to converge at the c-Src/p130Cas pathway, the PI3K/Akt pathway and, to a lesser extent, the NF-κB pathway. These observations are also consistent with the reported link of FAK, c-Myc and BRD4 to the inflammatory nature of breast tumor cells [44, 57–60]. Our findings support a close link between EMT and the inhibitor sensitivity of BRCA1/2 mutation-positive TNBC cells (i.e., HCC1937 and HCC1143). This unexpected observation is consistent with the known role of the EMT-inducing transcription factors Slug and Snail in the regulation of the cellular adhesion network, such as fibronectin and their receptors (e.g., α5β1 integrin) [61], and the link of BRD4 to FAK [62]. This notion is also corroborated by the role of BRD4 in cell differentiation [60, 63]. Combined, these data indicate that repression of the BRD4/c-Myc axis promotes tumor cell dependence towards the integrin/FAK-mediated signaling pathways, suggesting a synthetic lethality-like effect of the JQ1/VS-6063 combination.

### Cooperative role of FAK and the BRD4/c-Myc axis in the tumor microenvironment

The results from our VS-6063/JQ1-based in vivo analyses support a cooperative role of the integrin-FAK and BRD4/c-Myc axes in the TNBC tumor microenvironment (Fig. 7). This finding is consistent with the effect of other FAK inhibitors in inflammatory breast tumor cells (e.g., SUM149) and the 4 T1 cell line [20, 64]. The cooperative effect of these inhibitors in MDSC cells (Fig. 7) is in line with the strong role of the BRD4/c-Myc axis in the maintenance of immunity [40]. However, whether this alliance is confined to 8q24 deregulation-bearing TNBCs remains to be addressed.

## Conclusions

Our study demonstrates that c-Myc and FAK are frequently co-overexpressed in breast tumors and that this deregulation significantly correlates with a poor prognosis of TNBC patients. At the cellular level, the BRD4-c-Myc axis and the integrin-dependent pathways act cooperatively to drive tumor cell survival, growth and microenvironmental changes. These pro-tumorigenic activities are highly susceptible to JQ1/VS-6063-based co-targeting, and may serve as a key basis for the development of small molecule-based combinatorial targeted therapies for breast cancer.

## Electronic supplementary material

ESM 1 (PDF 417 kb)

ESM 2 (PDF 55 kb)

ESM 3 (PDF 22 kb)

ESM 4 (PDF 118 kb)

ESM 5 (PDF 1640 kb)

ESM 6 (PDF 19 kb)

ESM 7 (PDF 13 kb)

ESM 8 (PDF 41 kb)
