# Supplementary figures and images for: BRD4 modulates vulnerability of triple-negative breast cancer to targeting of integrin-dependent signaling pathways
Source: Cell Oncol (Dordr). 2020 Oct 2;43(6):1049–66. doi: 10.1007/s13402-020-00537-1 (PMC7716866; doi:10.1007/s13402-020-00537-1)

# Figure S1

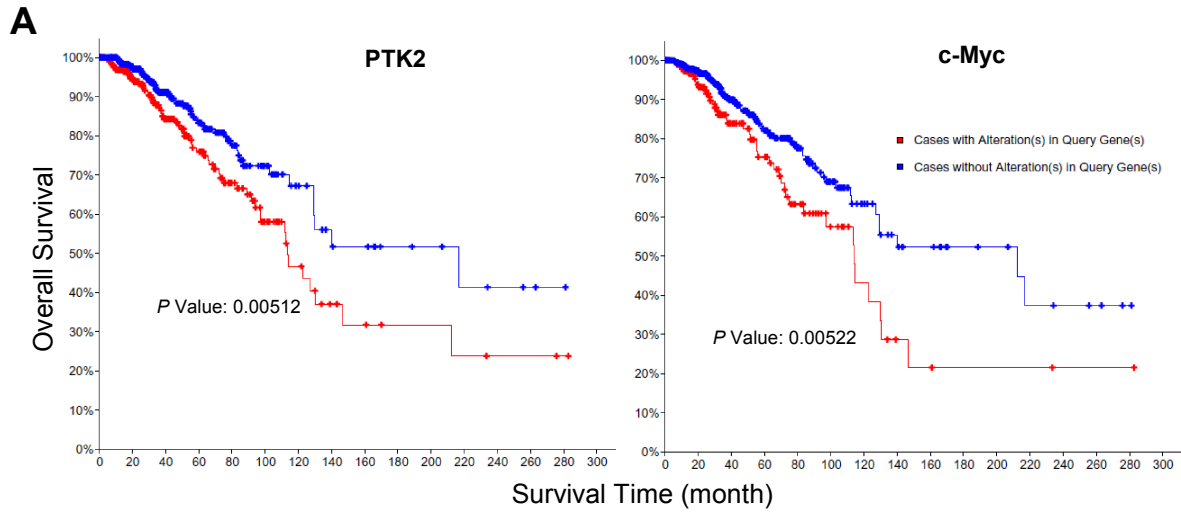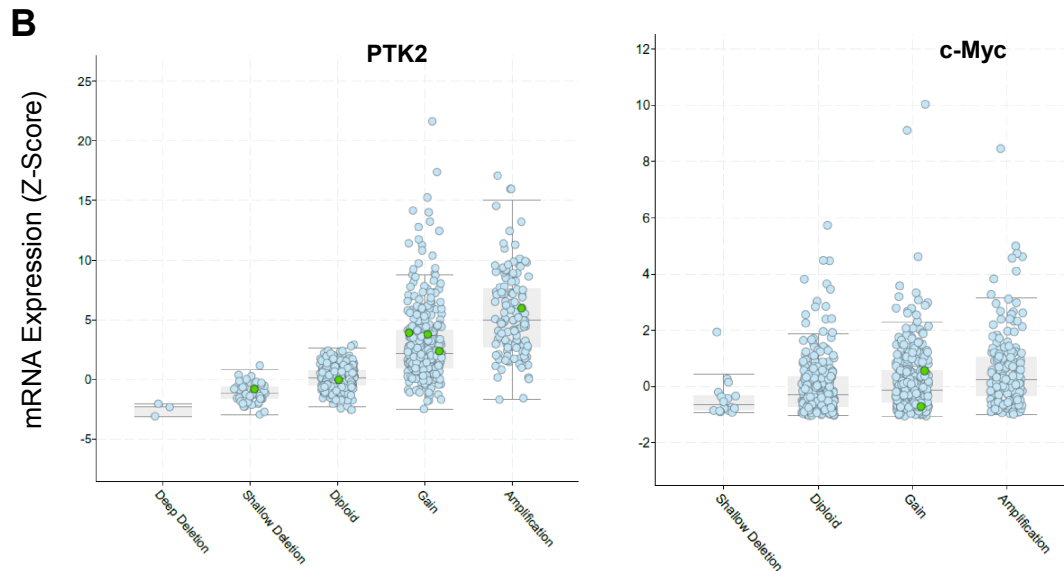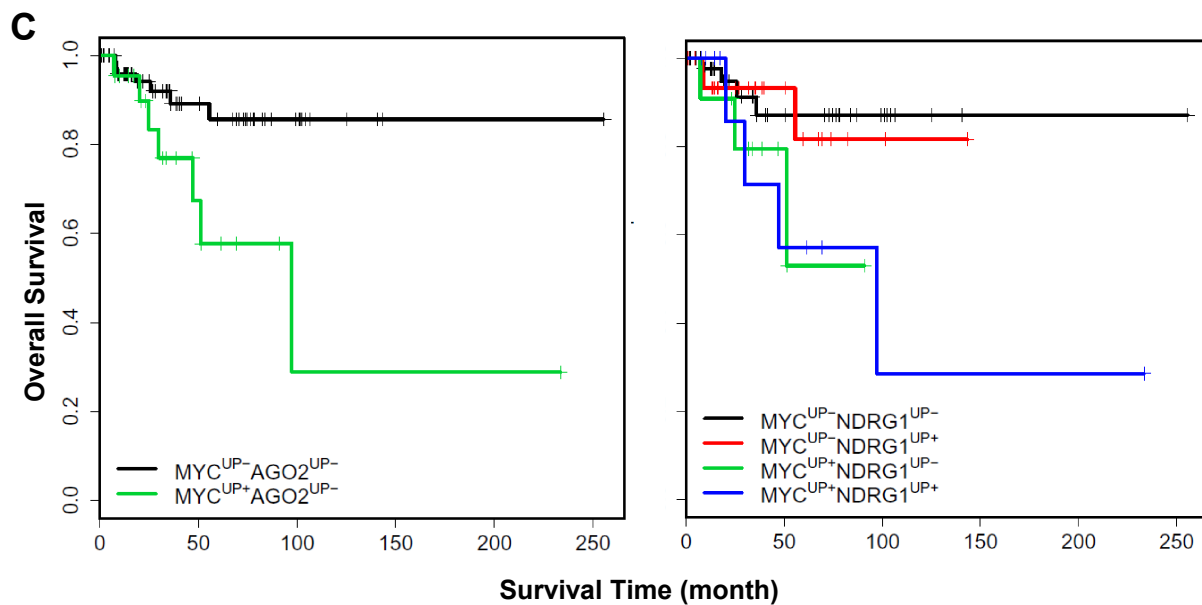

Supplement: Supplementary file 1 — (PDF 417 kb) [file 13402_2020_537_MOESM1_ESM.pdf]

Figure S2

Cell Viability (%)

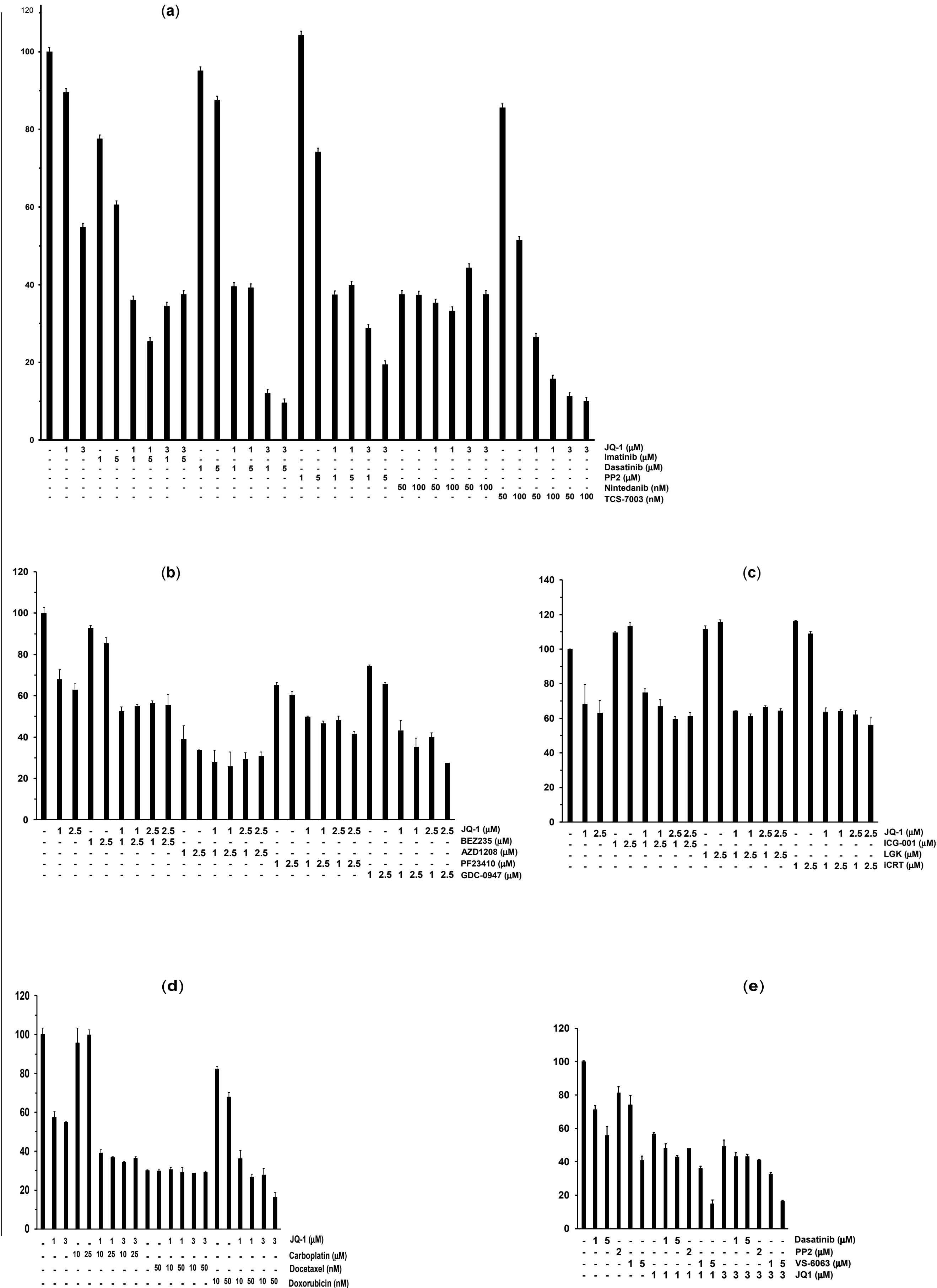

Supplement: Supplementary file 2 — (PDF 55 kb) [file 13402_2020_537_MOESM2_ESM.pdf]

Figure S3

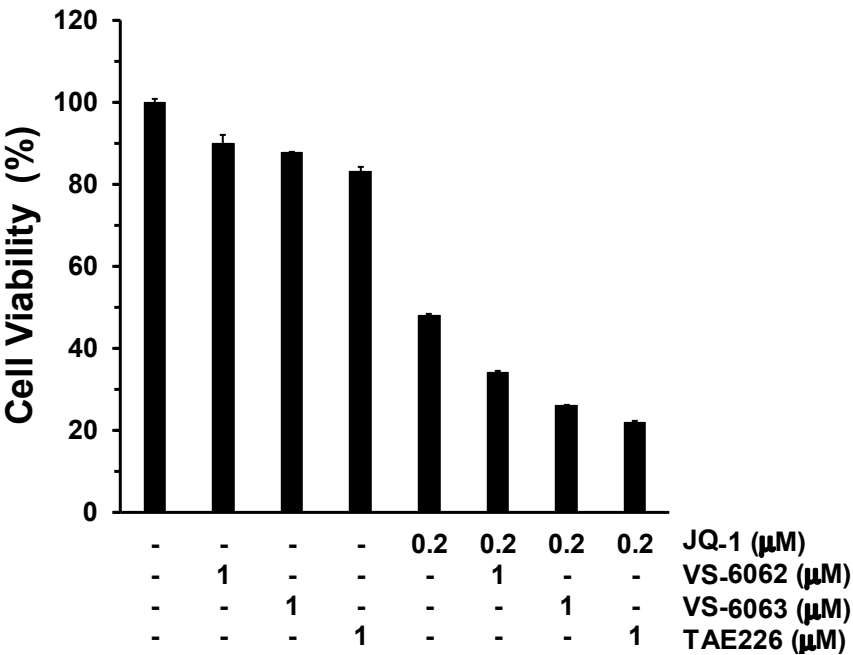

Supplement: Supplementary file 3 — (PDF 22 kb) [file 13402_2020_537_MOESM3_ESM.pdf]

Figure S4

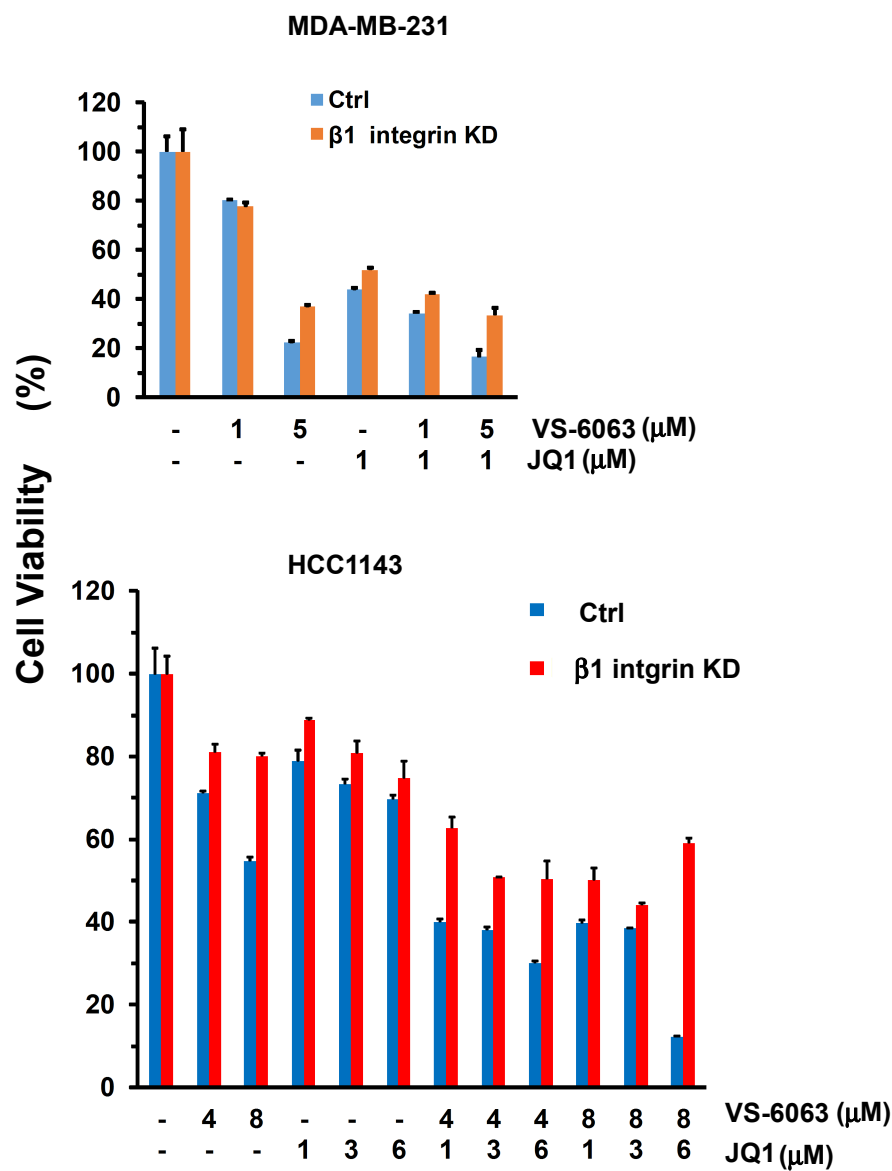

Supplement: Supplementary file 4 — (PDF 118 kb) [file 13402_2020_537_MOESM4_ESM.pdf]

**Figure S5**

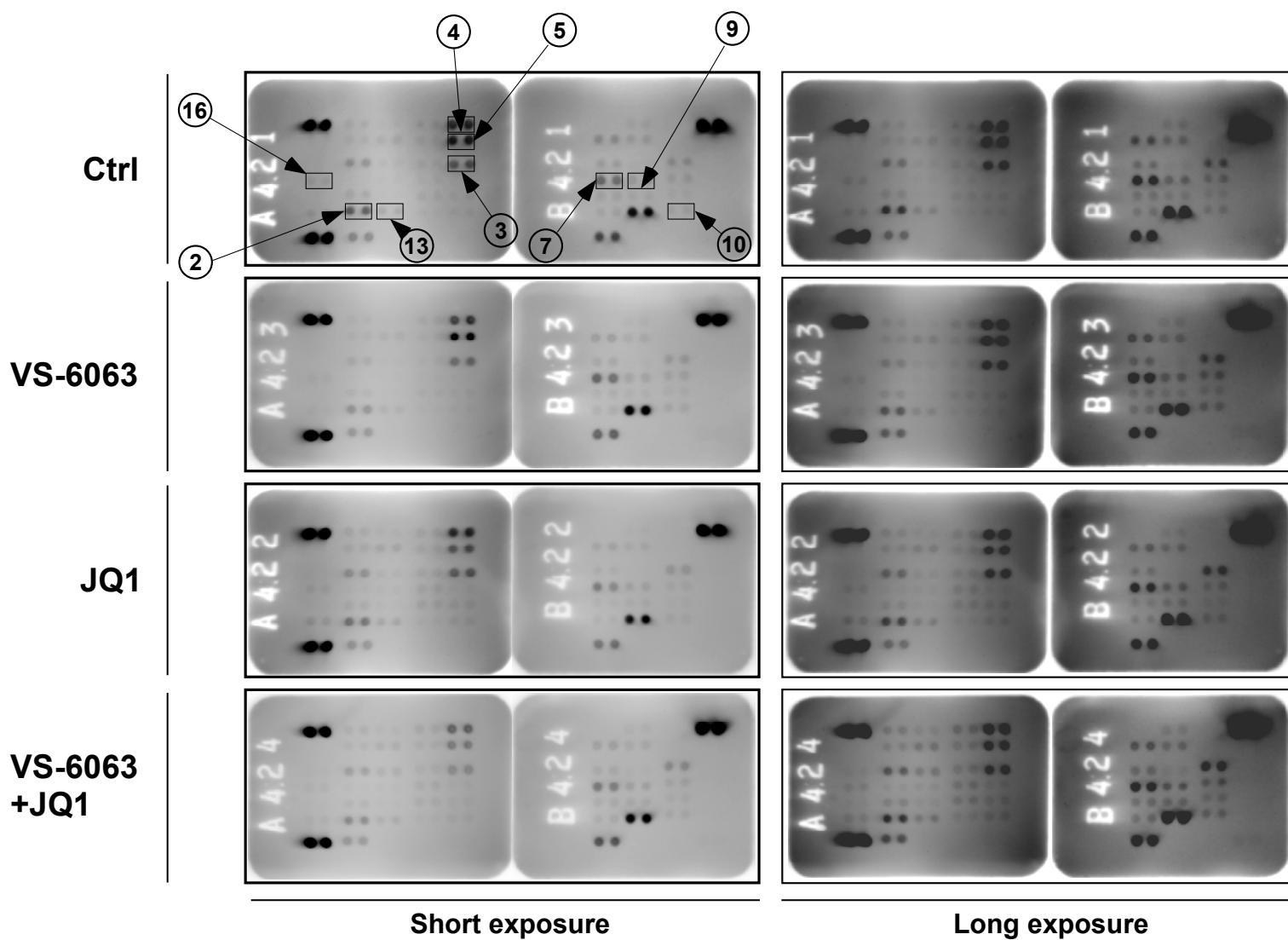

Supplement: Supplementary file 5 — (PDF 1640 kb) [file 13402_2020_537_MOESM5_ESM.pdf]
