# Supplementary material for: BRD4 modulates vulnerability of triple-negative breast cancer to targeting of integrin-dependent signaling pathways
Source: Cell Oncol (Dordr). 2020 Oct 2;43(6):1049–66. doi: 10.1007/s13402-020-00537-1 (PMC7716866; doi:10.1007/s13402-020-00537-1)
Supplement: Supplementary file 6 — (PDF 19 kb) [file 13402_2020_537_MOESM6_ESM.pdf]

Supplementary Table S1. Sources and sequences of siRNA oligos and gRNA

**SiRNA/gRNA**	**Sequence**	**Vendor**
BRD4	On-target plus – SMART pool	Dharmacon
Bim	GACCGAGAAGGUAGACAAUUG	CST*
	CCAACAGGAACUAUGACCUCGACUA	Genepharma
FAK	On-target plus – SMART pool	Dharmacon
Myc	On-target plus – SMART pool	Dharmacon
	GAGGACUUGUUGCGGAAACTT	CST
E-Cadherin	On-target plus – SMART pool	Dharmacon
β-catenin	GCACAAGAAUGGAUCACAATT	CST
β-catenin	GCUGAAACAUGCAGUUGUATT	CST
p65/RelA	On-target plus – SMART pool	Dharmacon
Snail	GGCACGTACCAGTGTGGGTC	
Slug	ATCTCTGGTTGTGGTATGAC	

*****: CST: Cell Signaling Technology
