# Supplementary material for: BRD4 modulates vulnerability of triple-negative breast cancer to targeting of integrin-dependent signaling pathways
Source: Cell Oncol (Dordr). 2020 Oct 2;43(6):1049–66. doi: 10.1007/s13402-020-00537-1 (PMC7716866; doi:10.1007/s13402-020-00537-1)
Supplement: Supplementary file 7 — (PDF 13 kb) [file 13402_2020_537_MOESM7_ESM.pdf]

Table S2.

**Tumor subtype** (No. patient)	**Cohort** (N=817)	**Her2** (N=120)	**Basal** (N=107)	**Luminal A** (N=201)	**Luminal B** (N=122)
**No. of tumors with amplification (%)** (FAK vs c-Myc)	129 (15.79) vs 173 (21.2)	27 (22.5) vs 38 (31.7)	32 (29.9) vs 51 (47.7)	21 (10.5) vs 26 (12.94)	33 (27.1) vs 39 (32.0)
**Spearman correlation**	0.73**	0.58*	0.68*	0.79**	0.77**
p value	<0.0001	<0.0001	<0.0001	<0.0001	<0.0001
**No. of tumors with mRNA upregulation (%)** (FAK vs c-Myc)	306 (37.5) vs. 53 (6.5)	49 (40.8) vs. 5 (4.2)	62 (57.9) vs. 24 (22.4)	62 (30.9) vs. 1 (0.5)	70 (57.4) vs. 4 (3.3)
**Spearman correlation**	0.073	-0.0035	0.14	0.11	0.065
p value	0.036	0.974	0.15	0.13	0.47

**: A strong correlation.

*: Intermediate correlation.
