# Supplementary material for: BRD4 modulates vulnerability of triple-negative breast cancer to targeting of integrin-dependent signaling pathways
Source: Cell Oncol (Dordr). 2020 Oct 2;43(6):1049–66. doi: 10.1007/s13402-020-00537-1 (PMC7716866; doi:10.1007/s13402-020-00537-1)
Supplement: Supplementary file 8 — (PDF 41 kb) [file 13402_2020_537_MOESM8_ESM.pdf]

Table S3.

TNBC Cell Line	Gene Amplification			Oncogenic Activation/Mutation		
	8q24 region	FAK	c-Myc	BRCA1/2	Ras/Raf	PI3K/Akt
**HCC1143**	√	√	√	√		
**HCC1937**	√	?		√		√
**HCC38**						
**HS578T**			√		√	
**HCC1806**	√	√	√	√		
**HCC1395**	√	√	√	√		
**SUM149**	√			√		
**SUM1315**			√	√		
**MDA-MB-157**						
**SUM159**	√				√	√
**MDA-MB-436**	√			√		
**MDA-MB-231**					√	
**BT549**	√	√	√			√
**4T1**	?	√	√	?	?	?

√: Being positive.

+: Indicate expression abundance or inhibitor effectivness.
